# Multiple sclerosis and primary vascular dysregulation (Flammer syndrome)

**DOI:** 10.1186/s13167-016-0062-6

**Published:** 2016-06-15

**Authors:** Katarzyna Konieczka, Simone Koch, Tatjana Binggeli, Andreas Schoetzau, Juerg Kesselring

**Affiliations:** 1 Department of Ophthalmology, University of Basel, Mittlere Strasse 91, CH-4031 Basel, Switzerland; 2 Department of Neurology and Neurorehabilitation, Rehabilitation Centre Valens, Valens, Switzerland

**Keywords:** Multiple sclerosis (MS), Flammer syndrome (FS), Primary vascular dysregulation (PVD), Questionnaire, Predictive diagnostics, Targeted prevention, Personalized medicine

## Abstract

**Background:**

Multiple sclerosis (MS) is an inflammatory demyelinating disease of the CNS of still unknown aetiology. Flammer syndrome (FS) encompasses a set of symptoms and signs that are primarily but not solely related to the dysregulation of blood vessels. The purpose of the present study was to determine whether FS symptoms occur more often in MS patients than in controls.

**Methods:**

Fifty-eight MS patients and 259 controls answered a questionnaire covering 15 symptoms and signs of FS.

**Results:**

Six of the 15 symptoms and signs of FS (dizziness, low body mass index, cold hands and/or feet, tendency toward perfectionism, reduced thirst, feeling cold) were found significantly more often in MS patients than in controls. Seven additional symptoms and signs (tinnitus, headaches, increased pain sensation, long sleep-onset time, migraines, increased response to certain drugs, low blood pressure) also occurred more often in MS patients, but the difference in frequency was not statistically significant. One sign (reversible skin blotches) was found less often in MS patients, but the difference in frequency was not statistically significant. One symptom (increased smell perception) was found significantly less often in MS patients.

**Conclusions:**

MS patients suffer significantly more often from FS symptoms and signs than controls. The reason for this association between MS and FS and the potential implications of this association still need to be determined.

## Background

Multiple sclerosis (MS) is a chronic, demyelinating, degenerative disease of the central nervous system. It is the most common neurologic disorder of young adults, with approximately 2.5 million individuals affected worldwide. The lesions are typically disseminated and lead to a wide variety of symptoms. The clinical course is usually fluctuating and can lead to severe and irreversible disability [1, 2]. The disease was first described more than 150 years ago [3]. Whilst we know that MS is an immune-mediated inflammatory and neurodegenerative disease, there is still controversy about its cause [4–7].

Since the first description of the disease by Charcot, vascular changes have also been repeatedly described as occurring more often in MS patients [8], particularly in the eye [9–11]. The inflammation also involves vascular and perivascular tissue and thereby influences brain perfusion. But systemic vascular changes have also been described. Most of these studies focused on atherosclerosis and its risk factors [12]. To the best of our knowledge, the prevalence of primary vascular dysregulation (PVD) [13, 14] (the vascular component of Flammer syndrome (FS) [15]), already described in eye diseases such as glaucoma [16, 17] and retinitis pigmentosa [18, 19], has not yet been studied in MS. The FS describes the phenotype of subjects with an inherited increased sensitivity and altered response pattern (particularly of the blood vessels) to a number of stimuli like coldness or emotional stress.

The purpose of this study was to investigate the frequency of FS-related symptoms and signs in MS patients compared to controls with the help of a multiple-choice questionnaire. Knowing that the frequency of the symptoms of interest may vary between nations [20], we compared Swiss MS patients with Swiss controls.

## Methods

### Participants

Fifty-eight MS patients at the Department of Neurology and Neurorehabilitation, Rehabilitation Centre Valens in Valens, Switzerland (34 women and 24 men; 16 relapsing-remitting, 28 secondary progressive and 14 primary progressive forms, diagnosed according to revised McDonald criteria, EDSS (Expanded Disability Status Scale) 2.5–8.5, average 5.58), anonymously filled out our questionnaire. The patients’ age ranged from 36 to 76 years (mean 44.7 ± 14.2). At the same time, 259 control subjects (150 women and 109 men) visiting shopping centres were recruited and asked to anonymously fill out the same questionnaire. The control subjects’ age ranged from 18 to 89 years (mean 51.4 ± 18.0). Both MS patients and healthy subjects received a questionnaire with the information that they can voluntarily and anonymously fill it out. We did not use inclusion or exclusion criteria in either group: the only difference between these groups was the presence or absence of MS. This study was not submitted to an ethics committee for approval before the work began, and the ethics committee cannot approve the study retrospectively. However, the Kanton St. Gallen Ethikkommission have indicated that if the project would have been submitted to the ethics committee prior to initiation, there would have been a high likelihood for ethics committee approval and that this should not preclude publication. All subjects completed the study without any complaints.

### Questionnaire

The questionnaire (Flammer Syndrome Questionnaire) consisted of 15 multiple-choice items with the following choices: ‘often’, ‘sometimes’, ‘never’ or ‘I do not know’. The questionnaire items are listed in Table 1.

**Table 1:** Symptoms and signs of Flammer syndrome

Cold hands or/and feet	
Reduced feeling of thirst	
Low blood pressure	
Dizziness	
Increased response to certain drugs	
Migraines	
Headaches	
Tinnitus	
Low body weight	
Feeling cold	
Long sleep-onset time	
Good smell perception	
Increased pain sensation	
Reversible skin blotches (red or white)	
Tendency toward perfectionism	

### Statistical analysis

In order to study the effect of the questionnaire items on MS patients compared to control subjects, a logistic regression analysis was performed, with each item used as a predictor. The most positive answer category was compared to the combined remaining answer categories (e.g. ‘sometimes’, ‘never’ and ‘I do not know’). Results are reported as odds ratios (ORs) and 95 % confidence intervals (CIs), with corresponding *p* values. Additionally, age, gender and a possible interaction between gender and each item were included in the regression models. A *p* value of <0.05 was considered significant. This study was exploratory; therefore, *p* values were not adjusted for multiple comparisons. All analyses were completed using R version 2.12.0 [21].

## Results

**Fig. 1:**
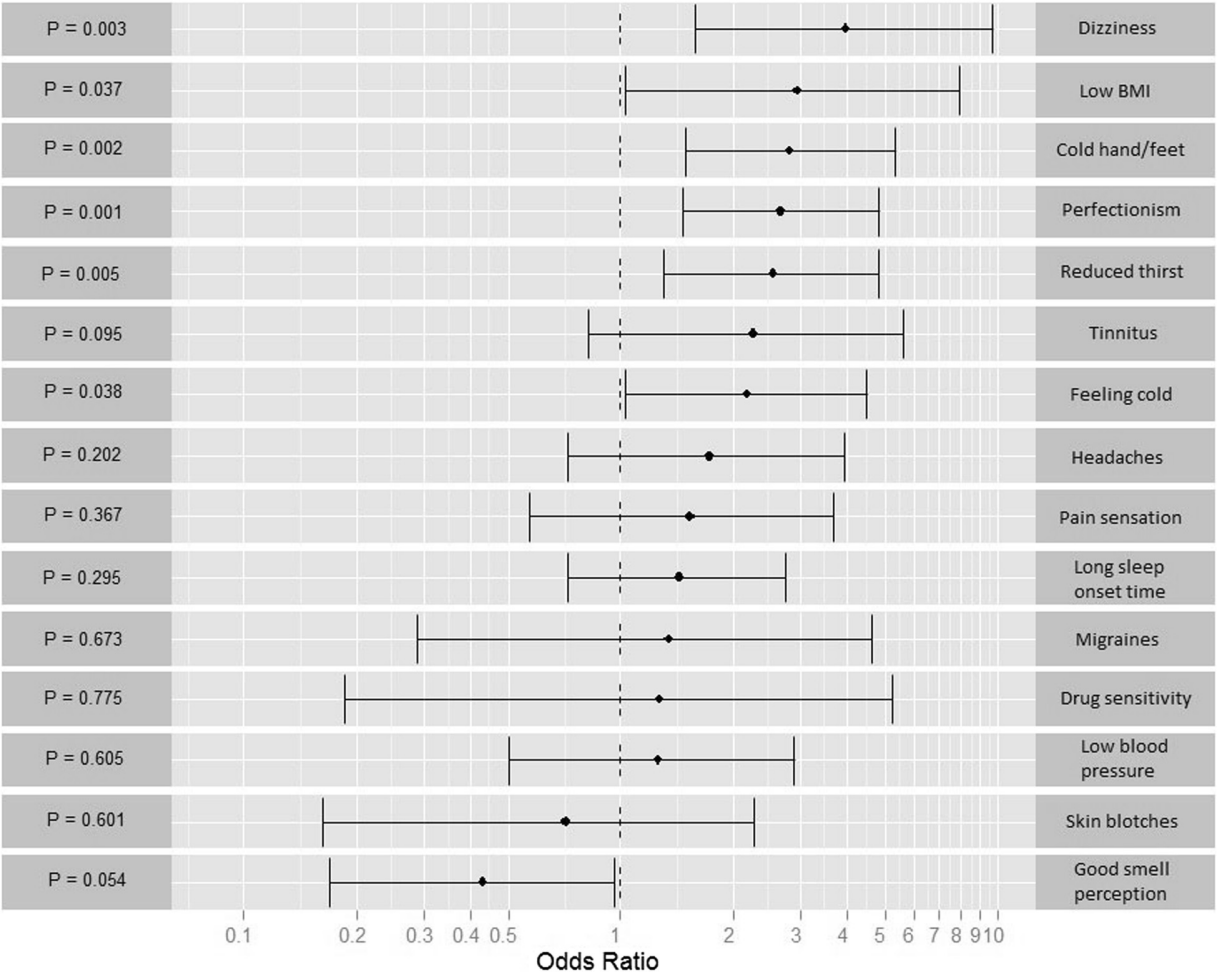
Frequency of symptoms and signs of Flammer syndrome in patients with multiple sclerosis (MS) (*n* = 58) in comparison to unselected controls (*n* = 259). For each of the questionnaire items listed in Table 1, results are presented as odds ratios (ORs) and 95 % confidence intervals (CIs), with corresponding *p* values. Results are sorted according to the degrees of difference between the two groups, beginning with the largest degree of difference. Ratios greater than 1.0 indicate that the symptom or sign occurs more often, and ratios less than 1.0 indicate that the symptom or sign occurs less often in MS patients than in unselected controls

Each questionnaire item was compared between MS patients and controls. The results are reported as odds ratios and sorted according to the degree of difference between the two groups, beginning with the largest degree of difference (Fig. 1). Ratios greater than 1.0 indicate a higher frequency of the symptom or sign in MS patients, and ratios less than 1.0 indicate a higher frequency of the symptom or sign in controls.

Six of the 15 symptoms and signs of FS were present significantly more often in MS patients than in controls. These were dizziness, low body mass index, cold hands and/or feet, tendency toward perfectionism, reduced feeling of thirst and feeling cold. Seven additional symptoms and signs also tended (although not statistically significantly) to occur more often in MS patients. These were tinnitus, headaches, increased pain sensation, long sleep-onset time, migraines, increased response to certain drugs and low blood pressure. One sign (reversible skin blotches) tended (although not statistically significantly) to occur less often in MS patients. One symptom (increased smell perception) was present significantly less often in MS patients.

ORs were adjusted for gender and age. No interactions between gender and any of the items were significant; therefore, interactions were not included in the regression models.

## Discussion

The present study reveals that 13 out of the 15 symptoms and signs characteristic of FS occur more often in MS patients than in controls. The difference in frequency was found to be statistically significant for six symptoms and signs and statistically insignificant for seven symptoms and signs. MS patients, however, indicated significantly reduced smell perception.

The findings of our study partly confirm previous reports, but they also reveal new information. Our MS patients suffer more often from dizziness than controls. Indeed, dizziness in MS has already been reported in the literature [22]. In addition, about 60 % of the MS patients have been reported to suffer from disequilibrium [23]. We found that our MS patients indicated more often to be slim than the controls. This is in agreement with previous studies. Compared to the general population, MS patients have lower rates of excess weight and obesity, as assessed by BMI. However, the literature is not homogenous. Some recent studies have shown that obesity (in particular adolescent obesity) might be a risk factor for MS [24, 25]. It is well possible that both extremes—very low and very high BMI—could increase the risk. Our findings show that MS patients subjectively suffer more often from cold extremities and more often feel cold than controls. This is also in agreement with previous reports. Thermoregulation dysfunction has already been described [26], but to the best of our knowledge, cold extremities in MS have not yet explicitly been reported in the literature. Our patients more often show perfectionist tendencies. Indeed, tendency toward perfectionism in MS patients has already been described [27]. Our patients have reduced feelings of thirst. This has not yet been described and may be due to increased endothelin-1 levels in the cerebrospinal fluid [28] or in the plasma [29]. Our results show (although statistically insignificantly) that MS patients more frequently suffer from tinnitus. Indeed, it has already been reported in the literature that 30 % of MS patients suffer from tinnitus [23]; even sudden hearing loss has been reported [30]. Our patients suffer more often (although statistically insignificantly) from headaches and migraines. Several previous studies have shown that headaches occur more frequently in MS patients than in controls or in the general population. Headaches may occur at the pre-symptomatic phase, at clinical onset and during the course of the disease [31]. Status migrainosus [32] or cluster headaches can occur as the initial presentation of MS. MS patients have been reported to suffer often from migraines [33]. Chronic pain is common among patients with MS. This is also in agreement with the results of our study. Our MS patients have a longer sleep-onset time. Sleep disturbance is also a common symptom of MS [34, 35]. Although increased odour perception is characteristic of FS, our MS patients indicate decreased smell function. Smell function is generally reduced in neurodegenerative diseases [36], and this has also been described in the MS literature [37].

Taken together, our results reveal a relationship between MS and FS. In addition to certain symptom-related similarities, the two entities share additional aspects, such as female preponderance [13], early onset and lower incidence in subjects with higher light exposure [13, 35, 38, 39]. Many lines of evidence suggest that higher serum vitamin D concentrations reduce the risk for both manifestation and progression of MS. MS and FS share further aspects, such as increased rigidity of the retinal vessels [40, 41], increased endothelin-1 plasma level [29, 42] and increased frequency of retinal gliosis-like alterations [13, 43, 44]. MS also shares some similarities with Susac’s syndrome, a vascular disease affecting the brain, retina and inner ear [45, 46]. Interestingly, patients with Susac’s syndrome often suffer from FS [47].

The phenomenology of FS has been described in detail elsewhere [13–15]. Already described is a relationship between FS and normal-tension glaucoma [16, 17], retinal vein occlusion, retinitis pigmentosa [19] and Susac’s syndrome, as recently summarized in a review [13]. To the best of our knowledge, this is the first report of a potential relationship between FS and MS.

The cause of this relationship remains at the moment unknown, and the impact of vascular dysregulation on the pathogenesis of MS remains open. There are several possibilities: MS could induce these symptoms (in terms of a secondary vascular dysregulation [13]), or subjects with FS may have a higher risk of developing MS. One could hypothesize that FS leads to clinically undetected microinfarctions in the central nervous system, triggering an autoimmune disease. FS also leads to increased oxidative stress [16], which in turn could contribute to the pathogenesis of MS [48–50]. A secondary vasculopathy (particularly endotheliopathy), induced by the autoimmune disease, could contribute to the chronic progression of MS.

### Limitations of the study

We did not match for age and gender; we did however include age and gender in the regression model. We also did not ask study participants for diseases except MS. MS is such a heterogenous disease that any sample has a potential bias. In a rehabilitation centre, patients with rather longer disease duration and higher EDSS than in the ‘average MS population’ are treated. Potentially confounding effects of immunomodulatory drugs cannot be excluded as they cannot be determined in the way this study was conducted. The study should be repeated with newly diagnosed MS patients without potential confounding comorbidities and treatments.

## Conclusions

MS patients suffer significantly more often from FS symptoms and signs than controls. Future studies will need to confirm the relationship between MS and FS and, if it is found to be positive, to analyse the cause of this relationship. FS is normally not treated in healthy subjects. However, if it turns out to be a risk factor for MS, prophylactic and preventive vascular treatment may be helpful. Such an individualized treatment could be based on the outcome of a vascular evaluation such as a dynamic retinal vessel analysis [51].

## Abbreviations

FS, Flammer syndrome; MS, multiple sclerosis; PVD, primary vascular dysregulation
